# Assessment of errors in death certification and mortality patterns and trends among medical patients at Korle Bu Teaching Hospital, 2019-2023

**DOI:** 10.4314/gmj.v59i3.2

**Published:** 2025-09

**Authors:** Francis Agyekum, Isabella E Asamoah, Muhyideen Bashir, Nana A Asante, Khushali Ganatra, Fiifi Duodu, Florence K Akumiah, David Brodie-Mends, Kofi T Asamoah, Eugene B Ampofo, Alfred Doku

**Affiliations:** 1 Department of Medicine and Therapeutics, University of Ghana Medical School, College of Health Sciences, University of Ghana, Accra; 2 Department of Medicine and Therapeutics, Korle-Bu Teaching Hospital, Accra, Ghana; 3 National Cardiothoracic Center, Korle-Bu Teaching Hospital, Accra, Ghana

**Keywords:** Medical Certification of Cause of Death, mortality data, noncommunicable disease, cardiovascular disease, errors, MCCD forms

## Abstract

**Objectives:**

This study audited the Medical Certification of Cause of Death (MCCD) forms issued at Korle-Bu Teaching Hospital, assessing their accuracy, completeness, and consistency while analysing trends in mortality causes.

**Design:**

Retrospective review of completed MCCD forms.

**Setting:**

Korle-Bu Teaching Hospital, Department of Medicine.

**Participants:**

All duplicate MCCD forms issued between January 1^st^, 2019, and December 31^st^, 2023, were included.

**Interventions:**

No intervention

**Main outcome measures:**

Frequency of major and minor errors in completing the MCCD forms and leading causes of death in the department.

**Results:**

Of 4,544 MCCD forms audited, 4,460 (98.17%) contained errors. Major errors were observed in 4,028 (88.64%) forms; the commonest being incorrect reporting of the underlying cause of death (3,483; 76.65%). Minor errors were nearly universal (99.82%), with omission of the full address of the deceased (4,530; 99.69%) being most frequent. Over half of the recorded deaths each year were attributable to non-communicable diseases (NCDs), with cardiovascular-related conditions being the leading cause of death.

**Conclusions:**

There was a high prevalence of errors in MCCD forms, highlighting the need for regular training of healthcare professionals to improve accuracy in death certification. Additionally, the high burden of NCD-related deaths emphasises the need to address modifiable risk factors, strengthen health systems, and foster multisectoral collaboration to mitigate the growing NCD epidemic.

**Funding:**

None declared

## Introduction

Mortality statistics are crucial in planning, evaluating, and monitoring healthcare delivery, interventions, and medical research.^1^ Therefore, accurate data is of utmost importance to aid policy decisions.

Medical certification of cause of death forms (MCCD) primarily provide documentation for medico-legal and administrative purposes.^2^ Bereaved families use MCCD forms as legal documents to claim end-of-life insurance and inheritance. They are valuable data for deriving key health indicators, such as specific mortality rates, which help monitor and evaluate health trends amongst populations. Additionally, statistical information from the MCCD data helps assess the effectiveness of public health programs/interventions/policies and informs future health policies and planning. Prioritisation of health and medical research programs and allocating limited health resources have been made possible by using statistical information derived from the analysis of MCCD forms.^3^

However, these data are useful only to the extent that health practitioners reliably certify the cause of death. Unfortunately, globally, there have been significant errors in death certification and its completeness, compromising its reliability for public health policy projection.^4^,^5^ These errors are broadly categorised into ‘major’ (errors that impact the selection of the underlying cause of death) and ‘minor’ (errors that have less effect on the classification of cause of death, though their absence affects precision).^6^,^7^ The most common mistakes are those that describe the mechanism of death rather than the underlying cause of death.^5^ The remote cause of death is often confused with the cause of death, and the events leading to death are improperly sequenced. Other errors include the non-documentation of time intervals, the use of unrecognised abbreviations, demographical errors, and the absence of the certifier's signature.^5^ In 2017, a study conducted in the Cape Coast Teaching Hospital in Ghana by Akakpo and colleagues revealed that nearly all of the MCCD forms for the year under review had major or minor errors.^8^ Similar findings were seen in a systematic review and meta-analysis by Alipour et al. on the common errors in death certification. The absence of a time interval was the most common error (80.9%), followed by the absence/inappropriateness of co-morbidities (45.1%) and the incorrect underlying cause of death (38.9%).^5^ Although some work has been done on the causes of death at multiple facilities within the Ghana Health Service,^9^ the Korle-Bu Teaching Hospital (KBTH), the national referral centre, was not one of these sites.

The World Health Assembly provides a standardised format for selecting a single cause of death for tabulation internationally through the International Classification of Diseases (ICD) cause of death codes.^10^ In that guide, the cause of death for primary tabulation should be the underlying cause of death (UCOD). The World Health Organisation (WHO) defines the UCOD as “the disease or injury which initiated the train of morbid events leading directly to the death.”^11^ The World Health Assembly has provided a standard MCCD form to certify the medical cause of death in all regions.

The medical practitioner signing the death certificate is responsible for indicating the UCOD and what conditions contributed to the death. Accurate completion of the MCCD form is imperative for obtaining reliable information on the medical causes of death. Hospital data on morbidity and mortality can help evaluate the standards of healthcare delivery, inform health priorities, and guide planning for health resource allocation.^12^ For instance, the causes of death, even in the WHO African region, over the past two decades have seen a gradual shift towards NCDs.

However, more than half of the deaths in the region have also been attributed to communicable diseases.^13^ Over 20% of hypertension in 1990 was undiagnosed in the African region by 2019, and adults aged between 30 and 79 years were estimated to have an age-standardised prevalence of hypertension of 38%.^14^ The global strategy has been the detection, screening, and treatment of these NCDs.^15^ If we know the causes of deaths, we can accurately shape the health services and policies we adopt and develop to address health needs.

We aimed to audit the MCCD forms filled out at the Department of Medicine and Therapeutics (DOMT), KBTH, from January 2019 to December 2023. The research questions were: What is the frequency of major and minor errors in completing the MCCD forms at the Department of Medicine and Therapeutics, KBTH? What proportion of deaths are from noncommunicable diseases (NCDs)? And what are the top ten causes of death in adults by sex?

## Methods

### Study design

From June 3rd to September 13th, 2024, a retrospective review of all duplicate MCCD forms issued from January 2019 to December 2023 was performed.

### Study setting

Korle-Bu Teaching Hospital (KBTH) is Ghana's premier national referral hospital, serving patients nationwide and from neighbouring countries. The DOMT is one of the hospital's largest departments, with four medical admission wards and four specialised wards: the chest clinic, stroke unit, renal unit, and the Korle-Bu Infectious Disease Centre. All death certificates are issued at a central office following consensus among nurses, residents, specialists, and consultants of the respective units and centre. The department manages over 450 inpatients and issues approximately 70-80 death certificates per month.

### Study population

The study included duplicates of all MCCD forms issued by the DOMT at KBTH between 1st January 2019 and 31st December 2023.

### Data collection and tools

The primary data source was the MCCD duplicate booklets in the custody of the Medical Directorate. Data was collected by five trained research assistants (medical doctors) and verified by two senior specialists. Permission was formally requested and granted to audit the forms and extract the required information. Data extraction was performed using an electronic data extraction sheet on Google Forms. The age, sex, and occupation of the deceased, as indicated on the form, were documented.

The UCOD was documented, as well as the other comorbidities contributing to the death. Patient names, folder numbers, and other personal identifiers were excluded to ensure anonymity. The disease classification was done using the International Statistical Classification of Diseases (ICD-11) categories.

The audit involved examining the completed form for accuracy, completeness of data, and logical reasoning regarding the cause of death. Errors on the completion of the form were classified as major or minor errors based on the following criteria.^8^

Major errors included:

- Not indicating the underlying cause of death on the MCCD form.
- Inaccurate sequence of events leading to death.
- Unrelated causal events stated as related.

Minor errors included:

- Omission of the address of the deceased or incomplete address.
- Date of last seen alive not stated.
- Time of death not stated.
- Duration of illness not stated.
- Use of ambiguous or confusing abbreviations.
- Manner of death not indicated.

Other errors on the form, excluding major errors.

Causes of death were further categorised into communicable/infectious diseases and noncommunicable diseases.

### Data analysis

The electronic form was extracted into Microsoft Excel, cleaned, and exported into STATA version 16 for analysis. Using simple tabulations, the socio-demographics (sex, age group, year of death), the top 10 causes of death with their respective categories, and the errors identified during the audit were described by proportions, and some were represented by pie, bar, or line graphs.

### Ethical considerations

Ethical approval for the study was obtained from the Korle-Bu Teaching Hospital Scientific and Technical Committee/Institutional Review Board with reference number KBTH-STC/IRB/00037/2024). The committee waived informed consent since the study involved a retrospective review of MCCD forms and no contact with any participants. Patient confidentiality and anonymity were strictly maintained. No patient identifiers were recorded on the data extraction form, and there was no direct contact with the relatives of the deceased patients, either in person or via telephone. The Google Form used for data extraction was password-protected to ensure data security.

## Results

### Socio-demographic characteristics of decedents

A total of 4,544 MCCD forms issued between 2019 and 2023 were analysed. The MCCD forms showed varying frequencies of missing data for each variable. The distribution of deaths varied across the years, peaking in 2021 at 23.12% (n = 1,003/4339). There were 787 deaths in 2019, 732 in 2020, 968 in 2022, and 849 in 2023. The year of death was not indicated on 205 MCCD forms.

The male-to-female mortality ratio was about 1.2, with 75% of decedents (n=3262/4343) dying before age 70, at a median age of 58 years (interquartile range: 45-69; range: 10-104). The ages of 201 decedents (4.42%) and the sexes of 104 (2.29%) decedents were not indicated on the MCCD forms.

### Frequency of major and minor errors in completing the MCCD form

Only one form was completed without errors among the 4544 MCCDs issued over the five-year period. Multiple errors were predominantly present (4,460; 98.17%). The majority (4,028; 88.64%) of the MCCDs had at least one ‘major error’, and almost all (4,533; 99.76%) had at least one minor error. The most common major errors were that the underlying cause of death was either incorrectly stated (3,483; 76.65%) or was not ticked (3,418; 75.22%). The most frequently observed minor errors were the incomplete documentation of the decedents' full addresses (4,530; 99.69%) and the omission of the duration of illness (4,173; 91.84%). (Table 1).

**Table 1:** Frequency of Major and Minor Errors in Completing MCCD Forms

Audit Parameter	Yes	No

	No. (%)	No. (%)
**Any Error (major or minor)**	4,543 (99.98)	1 (0.02)
**Single error**	83 (1.83)	
**Multiple errors**	4,460 (98.17)	
**Major Errors**		
**Is the sequence of events leading to death accurate and complete?**	3,254 (71.61)	1,290 (28.39)
**Is the underlying cause of death ticked?**	1,126 (24.78)	3,418 (75.22)
**Is the underlying cause of death correctly indicated?**	1,061 (23.35)	3,483 (76.65)
**Is an unrelated cause of event stated as related?**	371 (8.16)	4173 (91.84)
**Are there any major errors in the form?**	4,028 (88.64)	516 (11.36)

**Minor Errors**		
**Is the date last seen stated?**	4,532 (99.74)	12 (0.26)
**Is the date of death stated?**	4,339 (95.49)	205 (4.51)
**Is the manner of death indicated?**	4,175 (91.88)	369 (8.12)
**Has the full address of the deceased been provided?**	14 (0.31)	4,530 (99.69)
**Is the duration of illness stated?**	371 (8.16)	4,173 (91.84)
**Are there ambiguous abbreviations on the form?**	341 (7.50)	4,203 (92.50)
**Are there any minor errors in the form?**	4,536 (99.82)	8 (0.18)

Abbreviations and symbols: No = number of observations

**Figure 1:**
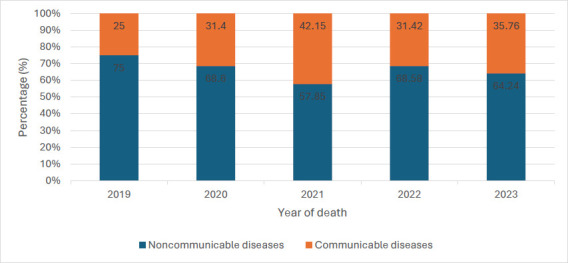
Proportion of Group Causes of Deaths by Year at the DOM, KBTH

Our analysis revealed that noncommunicable diseases accounted for the majority of deaths (n=2,153, 66.16%) during the study period. The proportion of deaths due to NCDs remained above 50% throughout the five years. However, the NCD death proportion declined by about 6.42% to 17.2% after 2019, with the highest drop seen in 2021 (Figure 1).

**Figure 2:**
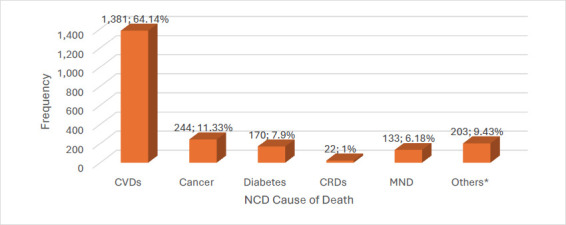
Frequency of major categories of NCD deaths between 2019-2023 at the DOM, KBTH CVD = cardiovascular disease, CRDs = chronic respiratory disease, MND = mental and neurological diseases, NCD = noncommunicable disease

Cardiovascular diseases (CVDs) accounted for most NCD deaths (64.14%), followed by cancers (11.33%) and diabetes-related deaths (7.90%) (Figure 2). Cancers included lung cancer (54), breast cancer (33), lymphoma (26), prostate cancer (26), multiple myeloma (24), leukemia (13), and others (68)) Noncommunicable chronic respiratory diseases, such as obstructive sleep apnea (10), idiopathic pulmonary fibrosis (7), bronchiectasis (3), asthma (1), and pulmonary sarcoidosis (1), accounted for only 1% of all NCD attributable deaths.

### Top ten underlying causes of death

**Figure 3:**
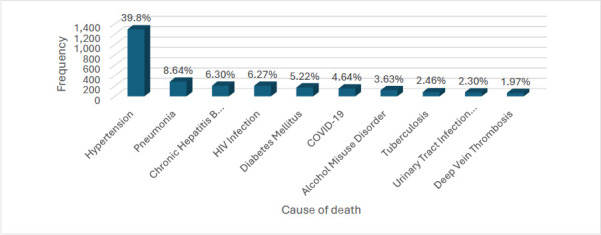
Top ten underlying causes of death among all decedents between 2019-2023 at the DOM, KBTH

The relative composition of the top six leading causes of death remained the same throughout the study period. Hypertension was the leading cause of death every year, contributing to about 40% of all deaths. Hypertension, pneumonia and diabetes consistently ranked among the top five leading causes of death annually (Figure 3). In 2019, HIV was the tenth leading cause of death; however, its death toll increased sharply after that year, making it the most common communicable disease and the second leading cause of death after hypertension in 2023.

**Figure 4:**
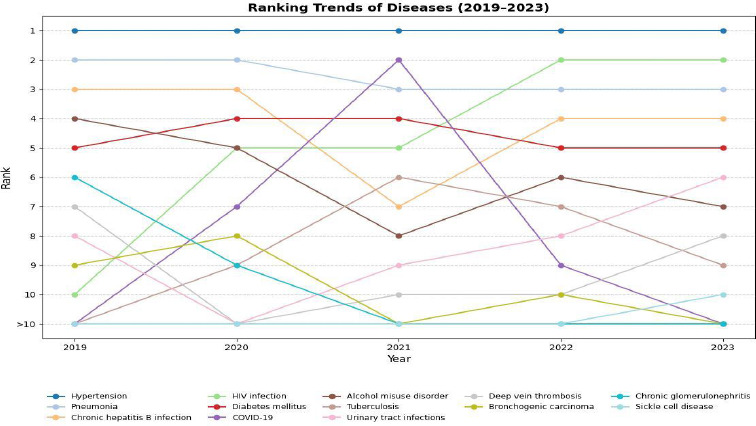
Trends in composition of causes of death, 2019-2023

**Figure 5:**
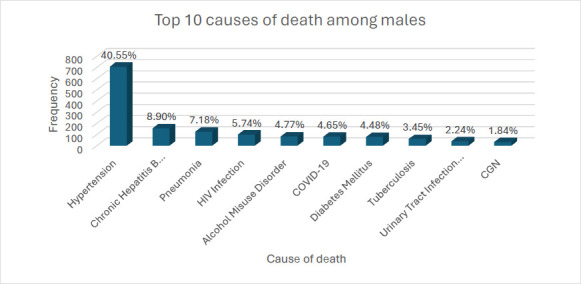
Top ten underlying causes of death among male decedents between 2019-2023 at the DOMT, KBTH CGN = Chronic glomerulonephritis, COVID-19 = Coronavirus 2019, HIV = Human immunodeficiency virus

**Figure 6:**
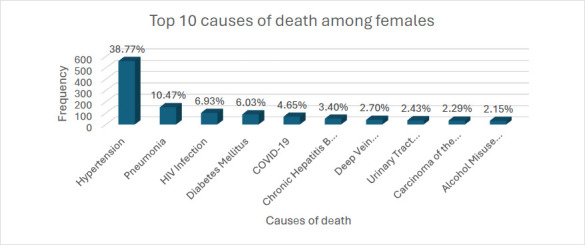
Top ten underlying causes of Death among female decedents between 2019-2023 at the DOM, KBTH COVID-19 = Corona virus 2019, HIV = Human immunodeficiency virus

COVID-19 emerged as a new infectious cause of death, with its share of deaths rising sharply and ranking as the second leading cause of death in 2021. However, its prevalence declined sharply, falling out of the top 10 causes of death by 2023 (Figure 4). There were sex differences in the top ten causes of death (Figures 5 and 6). Whereas hypertension was the commonest underlying cause of death for both sexes, chronic hepatitis B was among the top ten causes of death in males but not females. Alcohol misuse was also among the top 10 causes of death, with a higher proportion of deaths found in males (4.77%) than in females (2.15%).

## Discussion

This five-year departmental audit revealed a high prevalence of errors in MCCDs, with ‘minor errors’ being more common than ‘major errors’ and multiple errors being more common than single errors. Notably, major errors due to inaccurate documentation and/or marking of the underlying cause of death were found in over 88% of MCCDs, while minor errors were almost always present. The majority of deaths were premature, with males accounting for a higher proportion of deaths than females. More than three-quarters of decedents in our study suffered from a double burden of NCDs (mostly from cardiovascular causes) and CDs (mainly from HIV), contributing to their early deaths.

The overall error rate in this study was alarmingly high (99.98%), comparable to rates reported from studies in Iran (Jahanpour et al.), Nepal (Atreya et al.), India (Patil et al.), and Pakistan (Haque et al.).^8^,^16^-^19^

One consistent finding throughout these studies is that most of the MCCDs, if not all of them, originated from medical departments or that the majority of the deceased passed away from a circulatory or cardiovascular cause. In a prior study from Ghana, Ossei et al. purported that the fact that more than 50% of their MCCDs were error-free was the result of the education on death certification given to physicians during their medical training at their teaching hospital. A critical look at the study, however, showed that the quoted error rate was arrived at after the time interval of cause of death was excluded, given that the time interval of cause of death was missing from practically all of the MCCDs they reviewed.^20^

MCCDs frequently contain multiple major, minor, or both types of errors.^20^-^22^ Generally, minor errors are more common than major errors.^8^,^16^,^17^,^23^ Our study is consistent with this, with minor errors occurring in all but eight (99.82%) of the MCCDs issued during the years under review. By comparison, all the MCCDs in Akapko et al.'s study had minor errors.^8^ The omission of the patient's apparent or actual age in our analysis, in contrast to the study by Akakpo et al., where that parameter was observed to be the most common minor error, is a notable distinction that could explain the slight discrepancy in the frequencies of minor errors between the two studies. Another study in Ghana by Ossei et al. demonstrated a reversed pattern to both our findings and those of Akapko et al., reporting that only 7.4% of certificates had a single minor error. In contrast, a substantially higher 22.6% contained significant errors.^20^ One reasonable explanation for this discrepancy is that while “the use of abbreviations” was a minor error parameter shared by both studies, the key parameters that remarkably raised the minor error rates were neither considered nor analysed in either study.

The most common major errors found in studies are related to determining the underlying cause of death (UCOD).^24^ These errors have a major impact on accurately interpreting and appreciating disease trends and population health, which in turn influences the development of public health programs, the allocation of healthcare resources and the accuracy of public health surveillance. The significant error rate of 88.64% in this study was much higher than the results from other studies.^18^,^21^,^23^,^25^,^26^ Unfortunately, due to variations in the criteria used to evaluate significant errors in this study versus the other studies, it is difficult to compare the major error rates between them directly. For instance, our study reported a higher frequency of major errors (88.64%) compared to Akakpo et al.'s study (57.5%); however, the latter only employed two of the four parameters that we used for their major error criteria.

While improper sequencing errors were more prevalent in Akakpo et al.'s study,^8^ most of our major errors were either the result of failing to check the underlying cause of death or incorrectly documenting the cause of death (Table 3.2). Most patients receiving internal medicine care in our hospital are initially admitted to the emergency room before being moved to the medical wards, which include the medical intensive care unit. Their brief visits to the hospital's emergency room or intensive care units, where working diagnoses may be uncertain, could have resulted in challenges in determining the UCOD. Furthermore, certifiers must rely on the deceased's medical records for information when issuing a death certificate, which may be incomplete, particularly if the patient's examination before death was not sufficiently detailed due to a busy shift. A similar scenario was observed by Maharjan et al. in a study conducted in an ICU in Nepal.^24^ Additionally, medical patients frequently present as complex cases with multiple co-morbid conditions, making determining the UCOD more challenging. Eventually, certifiers who are unsure of the working diagnoses before a patient dies may resort to conjecture, misreport the cause of death, or employ ambiguous language, which can distort disease burden statistics and public health planning. In our department, residents are frequently assigned the task of writing death certificates. These residents may lack sufficient knowledge of the patient's history or may not have been directly involved with the patient's care, increasing the likelihood of errors. To prevent this, some medical teams hold post-ward-round discussions to standardise how death certificates are completed. However, even with these discussions, the details for completing the “Part 1” section of the certificate may not be adequately communicated, or there may be insufficient documentation of the agreed-upon causes of death. Compared to Akakpo et al.'s study, our findings show fewer sequencing errors, possibly due to the implementation of these team discussions.

Finally, residents' inattention, indifference, and lack of training in standard ICD death certification processes may have contributed to the errors. The latter is acknowledged as a global concern.^27^ Noncommunicable diseases (NCDs) accounted for 66% of the deaths in this study, a higher prevalence than reported in previous research in Ghana.^28^,^29^ Mboera et al. observed a significantly lower percentage of deaths due to NCDs (27.3%) in their 2006–2015 retrospective analysis of mortality data from 39 Tanzanian health facilities.^30^ In contrast, a study by Gumede et al. in 2018, reported a higher NCD death percentage of 72.1% among non-HIV patients who died while receiving medical care at a South African tertiary hospital in 2018. The differences in the patient composition of the studies, care settings- including levels of care- and the distinct time periods of the studies likely explain the variation in the percentage of NCD deaths between our study and the studies mentioned earlier.

Over the past two decades, the WHO African region has observed a consistent increase in the proportion of NCD deaths, but no more than 50% of deaths attributed to NCDs have ever been documented. The global trend depicts a contrary picture.^13^ Our findings align with the regional shift towards NCD preponderance, as demonstrated by the institutional prevalence noted above and the global trend. Our study also confirms that, of the four main NCD disease groups that cause deaths globally, cardiovascular diseases (CVDs) are the leading cause of death.^31^ Interestingly, in contrast to the global trend that ranks diabetes as the fourth most common cause of NCD deaths, our study ranked mental and neurological diseases as the fourth most common cause of NCD deaths, surpassing diabetes. This may partly be explained by the high proportion of deaths (3%) due to alcohol-related disorders. Alcohol has been identified as not only a CVD risk factor but also a behavioural risk factor for NCD deaths.

According to the 2023 global report on hypertension, hypertension is the leading preventable risk factor for CVDs and a “silent killer,” claiming over 10 million lives annually.^14^ In a recent review by Doku et al., Ghanaians with hypertension were over 3 times more likely to develop CVDs than Ghanaians without hypertension.^32^ The fact that hypertension was the primary underlying cause of death over the 2019-2023 study period was, therefore, not surprising. In our study, hypertension-related deaths accounted for 40.6% of male and 38.8% of female decedents, significantly higher than global averages for 2019, which were 18% for men and 20% for women, respectively. Furthermore, the male-to-female proportion of hypertension-related deaths in our study was 1.8% higher in males, which inversely contrasts global trends, where it was 2% higher in females.^14^ The prevalence of hypertension continues to rise globally, but significantly more in Low and Middle-income countries (LMICs), including Ghana, where risk factors such as unhealthy diets (eg, high sodium intake and low potassium intake), obesity or increasing BMI, alcohol consumption and physical inactivity are commonly observed.^33^,^34^ Enforcement of existing public health policies and the development of population-based strategies are necessary to reduce the prevalence of hypertension in Ghana.

Finally, it is important to note that the Department of Pathology already provides death certification training during final-year medical student rotations and resident training.

However, to improve outcomes, we suggest that greater emphasis be placed on assessing the acquisition and application of these skills during clinical training. Incorporating formal evaluations in both undergraduate and postgraduate assessments may enhance attentiveness to this critical area. Furthermore, periodic CPD sessions for junior doctors and medical officers would help reinforce best practices and reduce persistent documentation errors.

Given the status of hypertension and, subsequently, CVDs as the major drivers of NCD deaths, there is an urgent need for national and local policies to prioritise the prevention, detection, treatment, and control of hypertension. Specific population-based strategies are essential to mitigate this growing public health challenge and effectively reduce the burden of NCD.

### Limitations

While this study provides valuable insights, several limitations should be considered when interpreting the findings. In addition to the inherent limitations of a retrospective design, the review of only MCCDs from the Department of Medicine and Therapeutics limits the generalisability of the study's findings. There is no standardised definition of major and minor errors, and various studies have used slightly different definitions, making it difficult to compare the proportions. Most of the decedents did not undergo a post-mortem examination, so the determination of the cause of death based solely on medical records may have been inaccurate. Additionally, medical patients frequently have multiple co-morbidities, which complicates the selection of a single UCOD.

## Conclusion

The audit identified a significant occurrence of errors in MCCD forms, with minor errors occurring more frequently than major errors and multiple errors being more prevalent than single errors. This underscores the necessity for ongoing training of healthcare professionals to enhance precision in death certification. Furthermore, most deaths were premature, primarily resulting from a significant dual burden of noncommunicable diseases (predominantly cardiovascular) and communicable diseases (chiefly HIV). This highlights the necessity of addressing modifiable risk factors, enhancing health systems, and promoting multi-sectoral collaboration to mitigate the growing epidemic of non-communicable diseases.
